# Huge incisional hernia: A case report

**DOI:** 10.1186/1757-1626-1-202

**Published:** 2008-10-02

**Authors:** Ehab Elganainy, Alaa A Abd-Elsayed

**Affiliations:** 1 Urology department, Assiut University Hospital, Assiut University, Assiut, Egypt; 2 Public Health and Community Medicine Department, Faculty of Medicine, Assiut University, Assiut, Egypt

## Abstract

**Introduction:**

The incidence of incisional hernia depends on many factors factors including old age, sex, obesity, bowel surgery, suture type, chest infection, abdominal distension and wound infection.

**Case report:**

A 55 years old woman presented at out institute, she had an operation 19 years ago – elsewhere – to remove a branched stone from her right kidney and admitted for two months into the hospital at this time as she had troubles with her surgical wound and she had repeated secondary sutures. The hernia is in the right lumbar region, has a smooth surface, shows expansile impulse on coughing, there are some dilated veins on its surface, there is no tenderness, it has a uniform consistency, mobile, there are no pulsations, it contains large and small intestine, partially reducible, the defect is about 10 cm in diameter and there are no complications. This woman had left nephrectomy two years ago as a treatment for painful non functioning kidney and she is living now with only functioning right kidney with serum creatinine 1.1 mg % and blood urea 38 mg %.

**Conclusion:**

Incisional hernia is a very common complication of wound healing after surgery. Good care and precautions are very important to avoid its development.

## Introduction

Incisional hernias develop in 3.8 – 11.5% of cases after abdominal surgery. The incidence depends on a number of factors including old age, sex, obesity, bowel surgery, suture type, chest infection, abdominal distension and wound infection [1,2]. Ninety percent of incisional hernias occur within 3 years of operation [3]. Repair of large abdominal incisional hernias is a difficult surgical problem with recurrence being a common complication. Recurrence rates of up to 33% after first repair and 58% after second repair have been reported [4].

## Case report

**Figure 1:**
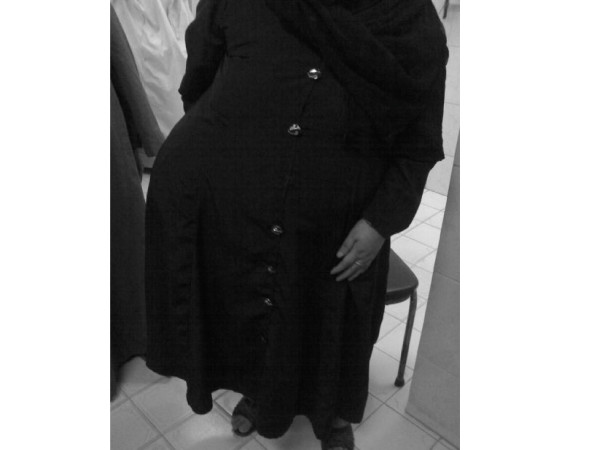
The patient at the time of presentation.

This is a 55 years old woman presented at our institute. We were so surprised at the first moment we saw this woman and we kept thinking what could be this bulge under her clothes (figure 1), we thought she is hiding a bag under her clothes as it is a habit for some Egyptian farmers to keep precious things in a bag or big wallet fixed to a belt under their clothes, but then as she started to tell us about her medical history we discovered that this a huge incisionl hernia.

She had an operation 19 years ago – elsewhere – to remove a branched stone from her right kidney and admitted for two months into the hospital at this time as she had troubles with her surgical wound and she had repeated secondary sutures. She had repeated blood transfusions during and after that operation.

**Figure 2:**
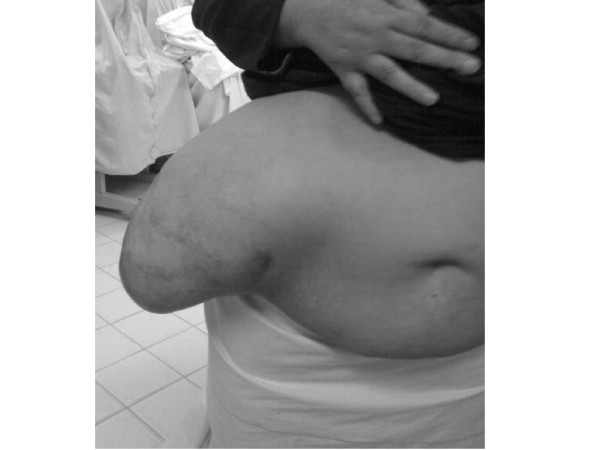
The hernia in the standing position.

**Figure 3:**
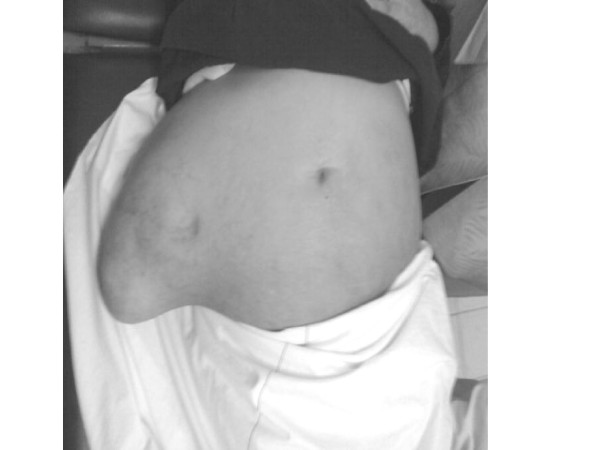
The hernia in the supine position.

The hernia is in the right lumbar region, has a smooth surface, shows expansile impulse on coughing, there are some dilated veins on its surface, there is no tenderness, it has a uniform consistency, mobile, there are no pulsations, it contains large and small intestine, partially reducible, the defect is about 10 cm in diameter and there are no complications (figure 2, 3).

This woman had left nephrectomy two years ago as a treatment for painful non functioning kidney (advanced hydronephrotic with immeasurable cortex with multiple stones inside) and she is living now with only functioning right kidney with serum creatinine 1.1 mg % and blood urea 38 mg %.

## Discussion

In as many as 1 in 3 abdominal wall closures, the fascial layer of the wound will fail to heal due to hemodynamic instability of wound contamination, especially in malnourished patients [5]. As a result, approximately 200,000 incisional hernia repairs are performed each year in the United States alone at a financial cost of nearly 2.5 billion dollars [6,7]. Nearly 4 million abdominal and pelvic operations performed each year in the United States, it is estimated that another 200,000 incisional hernias may be going unrecognized or untreated. Incisional hernias occur as the result of combined biomechanical failure in an acute fascial wound when considering the clinically relevant impediments to acute tissue repair together with the normal function of the abdominal wall to support increasing loads during the postoperative recovery period [8]. Acute fascial separation occurs early in the postoperative period, leading to the delayed clinical development of abdominal wall incisional hernias [9]. This phenomena occurs early in the trajectory of acute wound healing at a time when wound tensile strength is very low or absent (postoperative days 0–30). This occurs as during the earliest period of acute wound healing that the wound depends entirely on suture integrity to maintain abdominal wall closure. Simultaneously, most patients are recovering from their procedures and returning to increased levels of activity and placing increasing loads across the acute wound during its weakest phase. The most frequently identified clinical risk factors include a suboptimal closure technique, deep wound infections, malnutrition, perioperative hypotension, steroid use, and aortic aneurysm disease [5,10,11]. Normally, an acute fascial wound needs to pass through a complex series of well-orchestrated molecular and cellular events beginning with hemostasis and inflammation and leading through angiogenesis and fibroplasia until a provisional matrix is formed that is capable of resisting the distractive forces of the abdominal wall [12]. The end point of acute wound healing therefore is the nearest approximation of normal uninjured tissue structure and function. In the case of the abdominal wall, this means the timely reestablishment of an efficient load-bearing scar at the myofascial layer. Abnormal progression of the acute wound-healing trajectory impairs the recovery of wound tensile strength.[13] The mechanism of incisional hernia formation is most often attributed to early mechanical wound failure as a result of either to the pulling through of suture passed through adjacent wound tissue, too loose or too-tight suture placement, or suture failure all occurring at a time when wound tensile strength is essentially zero [8].

The use of different combinations of composite (polypropylene and e-PTFE) or resorbable prosthetic materials, of mesh with hydrophilic coatings and of mesh coupled with flaps can provide a solution, even in cases of abundant loss of abdominal wall substance, when adequate covering of the inner surface cannot be achieved with peritoneum or omentum [14,15]. Our patient declined surgery and preferred conservative management of the hernia with frequent follow up.

## Conclusion

Incisional hernia is a very common complication of wound healing after surgery. Good care and precautions are very important to avoid its development.

## Competing interests

The authors declare that they have no competing interests.

## Authors' contributions

EE participated in follow up, management of the patient and the manuscript writing; AAAE participated in follow up, management of the patient and the manuscript writing. All authors read and approved the final manuscript.

## Consent

Written informed consent was obtained from our patient for publication of this case report and the accompanying images.
